# Utilizing cell line-derived organoids to evaluate the efficacy of a novel LIFR-inhibitor, EC359 in targeting pancreatic tumor stroma

**DOI:** 10.18632/genesandcancer.184

**Published:** 2019-02

**Authors:** Bradley R. Hall, Andrew Cannon, Christopher Thompson, Bindu Santhamma, Alejandra Chavez-Riveros, Rakesh Bhatia, Hareesh B. Nair, Klaus Nickisch, Surinder K. Batra, Sushil Kumar

**Affiliations:** ^1^ Department of Biochemistry and Molecular Biology, University of Nebraska Medical Center, Omaha, NE, USA; ^2^ Department of General Surgery, University of Nebraska Medical Center, Omaha, NE, USA; ^3^ The Fred and Pamela Buffet Cancer Center, University of Nebraska Medical Center, Omaha, NE, USA; ^4^ Evestra Inc., San Antonio, Texas, USA

**Keywords:** pancreatic ductal adenocarcinoma, 3D model, pancreatic stroma, LIF, LIFR

## Abstract

Survival of pancreatic cancer (PC) patient is poor due to lack of effective treatment modalities, which is partly due to the presence of dense desmoplasia that impedes the delivery of chemotherapeutics. Therefore, PC stroma-targeting therapies are expected to improve the efficacy of chemotherapeutics. However, *in vitro* evaluation of stromal-targeted therapies requires a culture system which includes components of both tumor stroma and parenchyma. We aim to generate a cell line-derived 3D organoids to test the efficacy of stromal-targeted, LIFR-inhibitor EC359. Murine PC (FC1245) and stellate (ImPaSC) cells were cultured to generate organoids that recapitulated the histological organization of PC with the formation of ducts by epithelial cells surrounded by activated fibroblasts, as indicated by CK19 and α-SMA staining, respectively. Analysis by qRT-PCR demonstrated a significant downregulation of markers of activated stroma, POSTN, FN1, MMP9, and SPARC (*p*<0.0001), when treated with gemcitabine in combination with EC359. Concurrently, collagen proteins including COL1A1, COL1A2, COL3A1, and COL5A1 were significantly downregulated (*p* <0.0001) after treatment with gemcitabine in combination with EC359. Overall, our study demonstrates the utility of cell lines-derived 3D organoids to evaluate the efficacy of stroma-targeted therapies as well as the potential of EC359 to target activated stroma in PC.

## INTRODUCTION

Pancreatic cancer (PC) is the third leading cause of cancer-related mortality and is responsible for over fifty thousand deaths per year in the United States [1]. Shortly after 2020, PC will surpass both breast and colon cancer to become the second most common cause of cancer-related mortality, second only to lung cancer [2]. This high mortality is partly due to increased incidence and insignificant change in survival rates over the last few decades. Due to the lack of effective diagnostic markers, the majority of PC patients are presented with metastatic disease and only 20% of patients are candidates for surgical resection. The rest of the patients are offered definitive chemotherapy [3, 4]. However, the majority of chemotherapeutic regimens have demonstrated little efficacy in the treatment of PC. This poor performance of the therapeutic regimen is, in part, due to the dense desmoplastic stroma, which interferes with the delivery of chemotherapeutic agents and promotes cancer cell growth, stemness and immune suppression [5-9].

The desmoplastic response in PC is so intense that stroma often accounts for the majority of the tumor weight [10]. Studies have demonstrated that orthotopically implanted PC cells produce larger tumors when co-implanted with pancreatic stellate cells (PSCs) [11]. In light of this, more recent work has focused on characterizing and modulating pancreatic stroma to improve the delivery and efficacy of chemotherapeutic regimen. However, the *in vitro* systems routinely used to evaluate the efficacy of drug candidates lacks the stromal component, making it difficult to select stroma-targeting candidates for pre-clinical or clinical evaluation. The cellular complexity seen within pancreatic tumors is difficult to replicate, however, few studies have utilized PSCs or fibroblasts, the cells responsible for the desmoplastic response, to investigate the contribution of stroma in overall PC pathology [12-14]. Regrettably, no study so far has used complex *in vitro* stroma containing systems to evaluate the efficacy of stroma-targeted therapies.

Another layer of complexity is the three-dimensional (3D) organization seen in the tumors, which has been shown to significantly contribute to tumor biology. The 3D *in vitro* models such as tumor-derived organoids have been developed for several cancers, including PC that recreates some of the histological features of PC [15]; however, these organoids lack PSCs. Moreover, the development of an organoid system is time consuming, expensive, and requires tumor tissue derived from human or murine models, which are significant limitations for use of these models in large scale screening applications. Likewise, development and utilization of genetically engineered murine models are expensive, and require a long latency period from generation to the analysis of therapy response.

In response to this urgent need for a more effective model to recapitulate PC stroma, we set out to develop a novel cell line-derived 3D organoid model that would allow the evaluation of potential stromal-targeting therapeutics while alleviating some of the problems inherent to current models. Here, we describe our model and report the results of a first-in-class drug, EC359 that downregulates the expression of markers of activated stroma in PC. EC359 has been shown to competitively inhibit LIF receptor complex (LIFR) by occupying LIF-binding site (PCT: 10,053,485). LIF is a pleiotropic member of the IL-6 family of cytokines secreted as a soluble factor in the tumor microenvironment (TME) [16]. LIF signaling is mediated by the LIF receptor (LIFR) complex, constituted by LIFR and glycoprotein 130 (gp130) [16]. Recent investigations have implicated the role of JAK-STAT signaling and LIF-mediated activation of cancer-associated fibroblast (CAFs) in the deposition of desmoplasia and its associated mechanisms in multiple cancers, including PC [17-19]. LIF functions as a growth factor in pancreatic carcinoma cells and the crosstalk between tumor cells and fibroblasts confer pro-invasive properties, in part, mediated by LIF signaling [20].

## RESULTS

### Development of 3D organoid with stromal compartment

**Figure 1:**
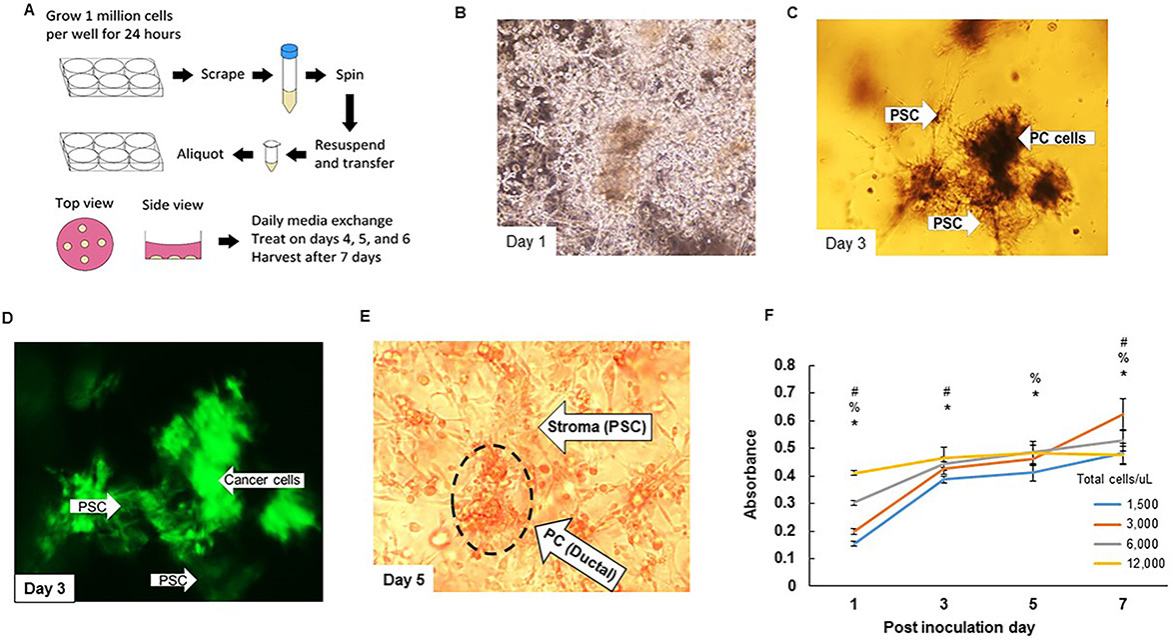
Pictorial representation of cell lines-derived 3D organoid **A.** Scheme of generation of cell lines-derived 3D organoids. Pancreatic cancer and stellate cells (FC1245 and ImPaSC at 1:2 ratio) are grown in monolayer culture for 24 hours at which point we scrape them, centrifuge them, and resuspended them in 1:1 mixture of matrigel: DMEM media supplemented with 10% FBS 1% Pen/Strep and aliquot in 30 μL increments. Cells are treated on PID 4, 5, and 6, and harvested on PID 7 for analyses. **B.-D.** (B) Microscopic view of organoids on PID 1 with little to no organization present. (C) Microscopic view on PID 3 with significant reorganization present. (D) Immunofluorescence demonstrating congregation of ductal cells. **E.** Microscopic view of organoids on PID 5 demonstrating the formation of organized structures within the organoids (Supplementary Figure 1, lower magnification, 200x). **F.** MTT assay demonstrating differential growth for varying cell densities. ^#^*p* < 0.05 for 3,000 *versus* 12,000, ^%^*p* < 0.05 for 3,000 *versus* 6,000, ^*^p < 0.05 for 3,000 *versus* 1,500.

Pancreatic cancer (FC1245, GFP expressing) and stellate (ImPaSC) cells were co-cultured together and subsequently seeded in matrigel (Figure 1A). The 1:1 ratio of Matrigel and media adequately maintained the 3D structures allowing for *in vitro* culture over the course of one week. Compared to tumor-derived organoids that require several growth factor supplements, we were able to grow cell line-derived organoids using DMEM media supplemented with 10% FBS. A total of 30 μl volume was adequate to plate and grow individual organoids. We noted that larger organoid volumes predisposed the organoids to shear-mediated disruption. First, the PC and stellate cells (ratio of 1:2) were seeded together in 6 well plate. After 24h, cells were scraped and mixed with matrigel: DMEM media, and seeded as organoids. We then followed the growth and organization of PC and stellate cells. On the post inoculation day (PID) 1, there was little to no organization and both cell types were indistinguishable and scattered in the matrigel (Figure 1B). By PID 3, there was a significant reorganization of the cells into distinct ductal and fibrotic structures as evident by phase contrast and immunofluorescence imaging of the GFP-expressing cancer cells (Figure 1C, 1D). On bright field microscopy, stellate cells demonstrated visible branching and interconnection with other cells, and by PID 5, we were able to demonstrate highly organized clusters of ductal and fibrotic structures within the matrigel scaffold (Figure 1E, Supplementary Figure 1).

### Growth kinetics of the cells in organoid

We next investigated the growth kinetics of the cells in the organoid using MTT assay. We standardized the initial seeding density of our cells to determine the linear growth rate of the organoids at varying plating densities over the course of seven days. The PC and stellate cells seeded in a 1:2 ratio, and plated at 3,000 total cells per microliter demonstrated continuous growth over the seven day period compared to cells plated at higher densities with growth rates that plateaued before one week and did not demonstrate any subsequent growth (Figure 1F). This initial seeding density of 3,000 total cells per microliter was chosen for all subsequent experiments.

### Characterization of the organoid

**Figure 2:**
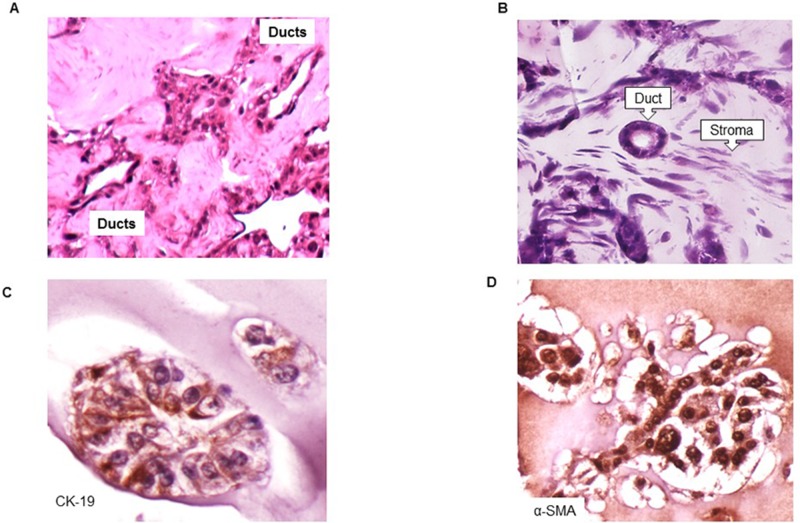
Organization of cells into ductal and stromal compartments in 3D organoids **A**. Hematoxylin and Eosin tissue staining depicting formation of pancreatic ducts adjacent to one another (Supplementary Figure 2A, lower magnification, 200x). **B**. Hematoxylin and Eosin tissue staining demonstrating a well-formed pancreatic duct surrounded by pancreatic stellate cells (Supplementary Figure 2B, lower magnification, 200x). **C.** Immunohistochemistry demonstrating pancreatic ductal cells stained using anti-CK19 antibody (1:40, TROMA-III, University of Iowa, IA). **D.** Immunohistochemistry demonstrating activated pancreatic stellate cells stained with anti-alpha-SMA antibody (1:500, ab7817, Abcam, Cambridge, MA).

After seven days of growth, we performed histologic analyses of cell line-derived organoids. Organoids were collected, fixed, embedded in paraffin blocks, and cut into 5 μm thick sections. Hematoxylin & eosin staining of the organoids consistently demonstrated the histology with the ductal and the fibrotic morphology (Figure 2A and 2B, Supplementary Figure 2A, 2B). Further, analysis with the ductal and activated fibroblast specific markers, CK19, and alpha-SMA, respectively, showed that the ductal structures demonstrated strong expression of the epithelial markers (Figure 2C). Moreover, we observed the varying levels of complexity in the ductal structures, possibly indicating the dynamic nature and evolution of the ductal structure in the presence of fibroblast compartment. Conversely, stellate cells adopted a spindle-like morphology in between the ductal cell structures (Supplementary Figure 2A, 2B), as seen in patient biopsy specimens, and these cells stained positive for alpha-SMA, indicating the activation of the PSCs (Figure 2D).

### Effect of gemcitabine and EC359 treatment on growth of organoids

**Figure 3:**
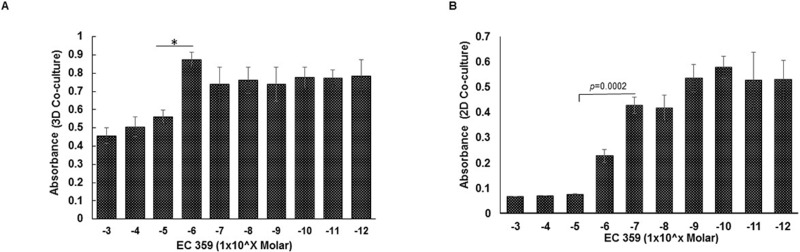
Comparative analysis of cellular response to EC359 treatment in 3D organoids and 2D co-culture **A.** The 3D *in vitro* MTT assay of organoids grown over the course of seven days and treated with varying concentrations of EC359 on PIDs 4, 5, and 6. MTT assay was performed on PID 7 demonstrating the IC 50 of 10uM for EC359 under *in vitro* conditions. B. The cells grown under 2D conditions were treated with EC359 for 72h and analyzed using MTT assay. ^*^p < 0.05.

We treated 3D cell line-derived organoids with the EC359, a specific inhibitor of LIFR, and gemcitabine followed by MTT assay (Figure 3A, Supplementary Figure 3). The effect of EC359 was also evaluated in cells grown in 2D co-cultures (Figure 3B). The 3D organoids were grown in 96-well V-bottom plates for seven days and treated with increasing concentrations of either gemcitabine or EC359 varying from 1 pM to 1 mM at PIDs 4, 5, and 6 (Figure 3A, and Supplementary Figure 3). Organoids were harvested for analyses on PID 7. The organoids were relatively more resistant to EC359 therapy compared to gemcitabine at equivalent concentrations (Supplementary Figure 3). The inhibitory concentration (IC50) for EC359 was approximately 10μM. For 2D analysis, cells were grown in 96-well plate and treated with EC359 for 72h. The cells grown under 2D conditions showed a greater response to EC359 compared to 3D culture with an IC50 of 0.7μM (Figure 3B).

### Effect on markers of activated stroma

**Figure 4:**
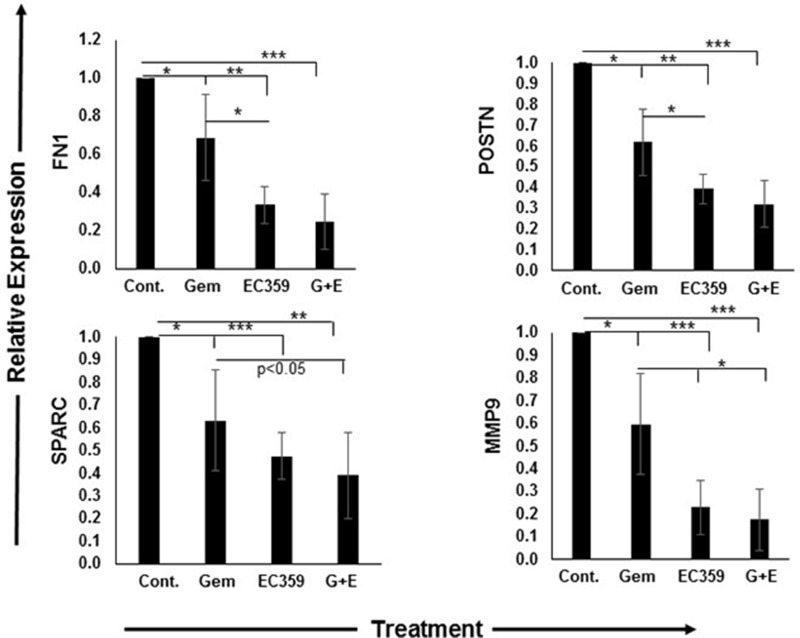
EC359 treatment significantly reduced the expression of activated stromal markers The qRT-PCR analyses of markers of activated stroma performed on 3D co-culture organoids. The treatment with EC359 (1μM), gemcitabine (1μM) alone and in combination (1μM each) resulted in higher downregulation of markers of activated stroma including FN1, POSTN, SPARC, and MMP9. *p < 0.01, **p < 0.001, ***p < 0.0001. Cont.: Control, Gem: Gemcitabine, G+E: Gemcitabine + EC359.

Next, we evaluated the ability of EC359 to modulate the activation status of pancreatic stellate cells in the organoids. Based on the previous studies on PC subtyping [10], we selected Periostin (POSTN), Fibronectin 1 (FN1), Secreted protein acidic and cysteine rich (SPARC), Matrix metalloproteinase 9 (MMP9) and Thrombospondin 2 (THBS2) as the markers of activated stroma as they are exclusively expressed by PC stroma with no contribution from the epithelial compartment, and also correlate with the poor prognosis in PC (Figure 4). Treatment with gemcitabine alone in 3D culture reduced the expression of POSTN, FN1, SPARC, and MMP9 by 38% (*p* < 0.01), 40% (*p* < 0.01), 37% (*p* < 0.01), and 31% (*p* < 0.01) at the transcript level, respectively (Figure 4, and Supplementary Figure 4A). Similarly, treatment with EC359 alone under 3D conditions also led to respective decreases of 61% (*p* < 0.001), 77% (*p* < 0.001), 53% (*p* < 0.0001), and 66% (*p* < 0.0001) at the transcript level, respectively (Figure 4). Combination treatment with both gemcitabine and EC359 led to the relative reduction in expression of POSTN, FN1, SPARC, and MMP9 were 68% (*p* < 0.0001), 82% (*p* < 0.0001), 61% (*p* < 0.001) and 68% (*p* < 0.0001), respectively, for cells grown as 3D organoid compared to untreated control. There was significant reduction in the expression of THBS2 (50%, *p* < 0.001) only in the presence of EC359. Treatment at the similar concentrations with gemcitabine and EC359 and their combination under 2D conditions showed a similar decrease in the expression of activation markers, however, there was significant toxicity that may have contributed to the higher decrease in expression of the markers of activated stroma (Supplementary Figure 5A, 5B).

### Effect on the expression of collagen protein

**Figure 5:**
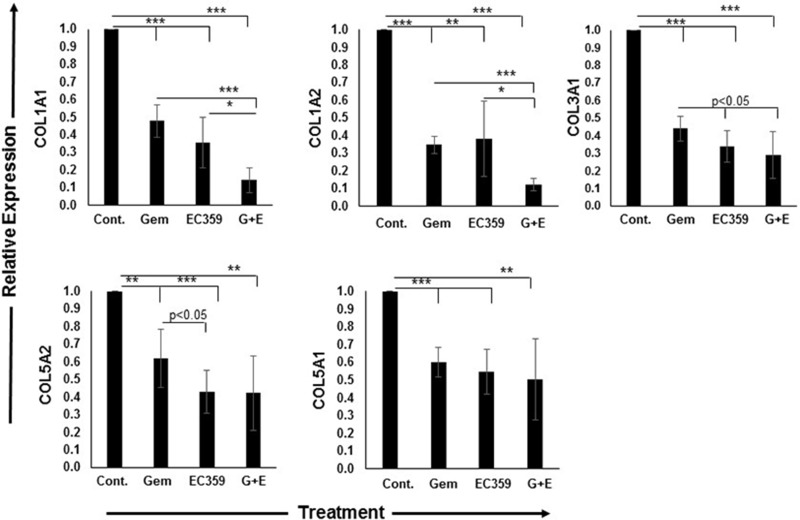
Collagen expression was significantly reduced after treatment with EC359 The qRT-PCR analyses of expression of various collagen performed on 3D co-culture organoids. The reduction in collagen expression in 3D model was significantly different across different treatments. Collagen expression including 1A1, 1A2, 3A1, 5A1, and 5A2 was significantly downregulated by EC359, and this effect was synergistic with gemcitabine. *p < 0.01, **p < 0.001, ***p < 0.0001. Cont.: Control, Gem: Gemcitabine, G+E: Gemcitabine + EC359.

Similar to activation markers, expression of various collagens is a hallmark of activated stroma, and also a major constituent of desmoplasia [10]. For all collagen markers, except collagen 10A1 (COL10A1) (Supplementary Figure 4B) and all treatment regimens including gemcitabine alone, EC359 alone, or both in combination, the relative reduction in collagen expression was more pronounced in 2D culture compared to 3D organoids (all *p* < 0.05). Treatment with gemcitabine alone in 3D culture reduced the expression of collagen 1A1 (COL1A1), COL1A2, COL3A1, COL5A1, and COL5A2 by 52%, 65%, 56%, 40%, (all, *p* < 0.0001) and 38% (*p* < 0.001) at the transcript levels, respectively (Figure 5). Treatment with EC359 alone led to respective decreases in expression of 64% (*p* < 0.0001), 62% (*p* < 0.001), 66% (*p* < 0.0001), 45% (*p* < 0.0001), and 57% (*p* < 0.0001), respectively (Figure 5). Following combination treatment with both gemcitabine and EC359, there was a more pronounced reduction in the expression of the aforementioned markers by 86% (*p* < 0.0001), 88% (*p* < 0.0001), 71% (*p* < 0.0001), 50% (*p* < 0.001), and 58% (*p* < 0.001), respectively, compared to untreated control (Figure 5). Similar to activation markers, equivalent concentrations of gemcitabine and EC359 under 2D conditions showed a similar decrease in the expression of collagen proteins and higher toxicity (Supplementary Figure 5A, 5B).

## DISCUSSION

The stroma in PC plays an important role in its pathogenesis. Existing data support the notion that targeting PC stroma will improve the delivery, and hopefully, the efficacy of the treatment regimens [21]. As a result, there is a significant need to create a useful *in vitro* 3D model to investigate the efficacy of therapeutics targeting PC stroma. Our novel cell line-derived organoid demonstrated that we are able to generate histological features of PC, including pancreatic ducts and associated stroma, using both PC and stellate cell lines. Additionally, we have characterized this model, optimized the methods such that it can be scaled up to screen multiple drug candidates targeting stromal compartment, and evaluated the expression of markers of activated stroma. A recent study attempted to subtype the PC on the basis of normal, tumor and stroma specific gene signatures. The tumor and stroma based subtyping classify PC into four subtypes, Basal-like with normal and activated stroma and classical-type with normal and activated stroma. The PC patients with the activated stroma showed poor survival with the unique expression profile characterized by the expression of SPARC, POSTN, FN1, MMP9, COL5A1, COL1A1, and COL1A2 genes [10]. The activated stroma is primarily derived from pancreatic stellate cells, which constitutes 4% of the total cellular component in the normal pancreas and proliferate extensively during PC progression. Various growth factors and cytokines activate PSCs, which in turn increase cancer cell proliferation, inhibit apoptosis, and promote epithelial to mesenchymal transition [16]. After activation, PSCs secrete extracellular matrix (ECM) proteins including collagen, fibronectin, hyaluronan, fibulin-2, and laminin. The cross-linked collagen fibers increase the stiffness and rigidity of tumor that directly participates in the integrin signaling and activation of other oncogenic molecules, including YAP1 [22, 23]. The stromal hyaluronic acid contributes significantly to the higher interstitial fluid pressure and poor tumor perfusion [21]. Therefore, we used this gene signature derived from the activated stroma to evaluate the ability of EC359 to target PC-associated fibroblasts.

Previous studies have demonstrated that EC359 has the potential to target fibroblasts in other tissues but the efficacy of this drug has never been investigated in PC (PCT: 10,053,485). Our study has demonstrated that EC359 can effectively and selectively decrease the expression of markers of activated stroma in PC. Further, we believe that the greater decrease in the expression of indicated genes in our study by the combined treatment with gemcitabine and EC359 is in part, contributed by the cytotoxic effects of gemcitabine. Therefore, it is reasonable to believe that EC359 could potentiate the effects of standard-of-care in PC. EC359 is believed to function by inhibiting LIFR and competes with LIF for binding to the ligand-binding site (PCT 10,053,485). However, the mechanism of action of EC359 warrants further investigation, especially for its use in PC. LIF is a pleiotropic member of the IL-6 family of cytokines and LIF binding to LIFR complex activates the JAK/STAT pathway. LIF and LIFR are widely expressed in many solid tumors and their overexpression is often associated with poor prognosis of patients [24]. LIF stimulates both growth and stemness in PC cells thereby potentially contributing to local growth, therapy resistance and distant metastasis [16, 25, 26]. The crosstalk between tumor cells and fibroblasts confers pro-invasive properties and LIF is shown to mediate the pro-invasive activation of fibroblasts.

Gemcitabine was approved for use in PC as the first-line therapy in 1997. Still, no drug is currently recommended to specifically target the pancreatic stroma. The combination regimens have shown better therapeutic outcome compared to a single agent, therefore, it is rational to believe that combinations that include a stromal targeting agent may significantly improve the efficacy of standard of care in PC [4, 27, 28]. Recent studies have renewed interest in the stroma-targeted therapies including enhanced drug delivery and inhibition of PSC-mediated chemoresistance. *Nab*-Paclitaxel in combination with gemcitabine showed a significant survival benefit in Phase III clinical trial (MPACT) by targeting desmoplastic reaction, and intratumoral accumulation of chemotherapeutics [29]. Additionally, it has been demonstrated that angiotensin II receptor blockers, Losartan modulate the activity of activated PSCs, inhibit their migration, and induce apoptosis thereby producing anti-fibrotic effects in PC [30]. PEGPH20, a pegylated hyaluronidase, was shown to have the therapeutic advantage in mouse models of PC through the modulation of the ECM resulting in increased tumor perfusion [21]. The JAK2 inhibitor, momelotinib is under investigation as an adjunct therapy to *nab*-paclitaxel and gemcitabine combination in Phase III, randomize, double blinded clinical trial (clinical trial identifier: NCT 02101021). Another JAK2 inhibitor, ruxolitinib, however, failed to demonstrate the therapeutic advantage in phase III clinical trial [31]. Neither trial of JAK2 inhibitor, however, attempted the concurrent targeting of the pancreatic tumor stroma and cancer cells. On these lines, the findings from our study are significant as treatment with EC359 in combination with gemcitabine may simultaneously target PC stroma as well as the cancer stem cells.

While our results are promising, it is important to address the limitations of our model. The cell line-derived organoid in our study has demonstrated efficacy against markers of activated stroma; however, it does not fully represent the entire cellular complexity of the tumor microenvironment seen in PC. Another limitation of our model is the ability to directly measure collagen protein. Matrigel is largely composed of collagen, and as such, our ability to measure PSC-derived collagen proteins is limited. However, it should be noted that matrigel contains little to no RNA, and as such, our qRT-PCR assays are of more utility.

## MATERIALS AND METHODS

### Cell culture and generation of 3D organoids

The murine PC and stellate cell lines, FC1245, and ImPaSC were generously provided by Dr. David Tuveson and Dr. Raul A. Urrutia, respectively. Both cell lines were cultured in 10% Dulbecco's Modified Eagle Media (DMEM) containing 4 mM L-Glutamine. Media was supplemented with 10% fetal bovine serum (FBS) and 1% penicillin/streptomycin. Cell lines were cultured in a humidified atmosphere containing 5% CO_2_ at 37°C. Prior to plating organoids, FC1245 and ImPaSC (1:2 ratio) cells were seeded in a 6-well plate at a density of 1×10^6^ cells per well and grown in monolayer culture. After 24 hours of growth, cells were scraped, spun down, and resuspended in 1:1 Matrigel: culture media solution such that cells were at a density of 3,000 cells/μL, assuming that both cells types doubled once in the 24 hour period.

### Cell viability

Cell viability in untreated cells was determined using the MTT assay. Cells at varying densities of 1,500, 3,000, 6,000, and 12,000 total cells/μL, 5 μL of 1:1 Matrigel: culture media containing cells were aliquot to wells in a clear 96 well V-bottom plate. Cells were grown at 37°C for seven days. On the seventh day of incubation, media was removed, and cells were incubated in 100 μL of MTT (10% MTT [5 mg/mL stock], 0.5% FBS, 89.5% serum free media) for 4 hours at 37°C. Formazan crystals were dissolved with 200 μL of dimethyl sulfoxide (DMSO), and the plate was agitated on a plate rocker for 30 minutes to allow for adequate dissolution of crystals in the 3D pellet. Then 100 μL was transferred to a clear 96-well flat bottom plate and absorbance was read at 595 nM on a 96 well plate-reader.

### Immunohistochemistry (IHC)

Immunohistochemistry was performed on 5 μm-thick, 4% paraformaldehyde-fixed paraffin-embedded sections [32]. The organoid sections were baked at 58°C for 8 hours and deparaffinized with xylene, and rehydrated in graded ethanol. Endogenous peroxidase activity was blocked with 3% H_2_O_2_, and antigens were retrieved by heating slides in citrate buffer at pH 6.0 for 15 minutes. Slides were washed, and blocked with horse serum for 1 hour at room temperature. Slides were incubated with mouse anti-alpha-SMA (1:500, ab7817, Abcam, Cambridge, MA) or anti-CK19 (1:40, TROMA-III, University of Iowa, IA) antibodies overnight at 4°C. The next day, slides were washed with PBS and incubated with HRP conjugated-Anti-mouse/rabbit antibody (Vector Laboratories, Burlingame, CA) in PBS for 1 hour at RT. Antibody binding was visualized using 3, 3′-diaminobenzidine (Vector Laboratories, Burlingame, CA) for 5 min at RT. Slides were counter stained with hematoxylin, washed, and dehydrated prior to mounting.

### Half-maximal inhibitory concentration (IC 50)

The half maximal inhibitory concentration (IC 50) was determined by seeding 15,000 total cells (1:2 ratio of FC-1245 to ImPaSC) in 5 μL of 1:1 Matrigel: culture media in replicates of eight in a 96 well plate. Media was changed twice daily for seven days. Cells were treated with either gemcitabine or EC359 at concentrations that varied by a factor of ten (1 pM to 1 mM). On the seventh day of incubation, media was removed, and cells were incubated in 100 μL of MTT (5 mg/mL, 10% MTT, 0.5% FBS) for 4h at 37°C. Formazan crystals were dissolved with 200 μL of DMSO, and the plate was agitated for 30 minutes to allow for adequate dissolution of crystals. Then 100 μL solution was transferred to a clear 96-well flat bottom plate and absorbance was read at 595 nM on a 96 well plate ELISA reader.

### Quantitative real-time PCR analysis (qRT-PCR)

Total RNA was isolated using a Qiagen kit (Qiagen, Germantown, MD) and used as a template for complementary DNA (cDNA) synthesized using 1 μg of RNA [32]. The expressions of various genes were profiled using gene specific primers (Table 1) as described previously.

**Table 1:** Primers pairs for the genes associated with activated stroma

Genes	Forward primer (5′>3′)	Reverse primer (5′>3′)
SPARC	GGATGTGGGCTTTTTCCCCT	TTGCCATGTGGGTTCTGACT
COL1A2	ACACCCTGACACCTGTTGTG	GTGGTGCGAATGTTCATGGG
COL3A1	TGTGGACATTGGCCCTGTTT	TGGTCACTTGCACTGGTTGA
POSTN	AGGTGGCGATGGTCACTTAT	TGGCCTCTGGGTTTTCACTG
COL5A2	TTGCCATCCCACAAAGCAGA	CCCACCAGGTTTTACGTGGA
COL1A1	TCTCCCCCAAGACACAGGAA	GGTAGGGAAGTAGACGGGGT
THBS2	TGAACTCGGCTGCAGTAAGG	TGGCCAAGTAAGAACTGCGT
FN1	ACGGTTTCCCATTACGCCAT	AAGCACTGGCATGTGAGCTT
COL10A1	AGGGAGTGCAATCATGGAGC	AGGACGAGTGGACGTACTCA
COL5A1	ATTCCAGGCCAAACGGTACAT	GGGGAGAAGTTAAAATCTGAGGC
MMP9	CATTCGCGTGGATAAGGAGT	TCACACGCCAGAAGAATTTG

### Statistical analysis

Student *t*-tests and ANOVA were performed to calculate the statistical significance and *p* < 0.05 was considered significant.

## SUPPLEMENTARY FIGURES



## Abbreviations

PDAC: pancreatic ductal adenocarcinoma
3D: three-dimensional
PBS: phosphate-buffered saline pH 7.4
TBS: Tris-buffered saline pH 7.6
TBST: 0.1% Tween 20 tris-buffered saline solution pH 7.6
IC 50: half maximal inhibitory concentration

## References

[1] Siegel RL, Miller KD, Jemal A (2017). Cancer Statistics, 2017. CA Cancer J Clin.

[2] Rahib L, Smith BD, Aizenberg R, Rosenzweig AB, Fleshman JM, Matrisian LM (2014). Projecting cancer incidence and deaths to 2030: the unexpected burden of thyroid, liver, and pancreas cancers in the United States. Cancer Res.

[3] Nitecki SS, Sarr MG, Colby TV, van Heerden JA (1995). Long-term survival after resection for ductal adenocarcinoma of the pancreas. Is it really improving?. Ann Surg.

[4] Hall BR, Cannon A, Atri P, Wichman CS, Smith LM, Ganti AK, Are C, Sasson AR, Kumar S, Batra SK (2018). Advanced pancreatic cancer: a meta-analysis of clinical trials over thirty years. Oncotarget.

[5] Binkley CE, Zhang L, Greenson JK, Giordano TJ, Kuick R, Misek D, Hanash S, Logsdon CD, Simeone DM (2004). The molecular basis of pancreatic fibrosis: common stromal gene expression in chronic pancreatitis and pancreatic adenocarcinoma. Pancreas.

[6] Sato N, Maehara N, Goggins M (2004). Gene expression profiling of tumor-stromal interactions between pancreatic cancer cells and stromal fibroblasts. Cancer Res.

[7] Koenig A, Mueller C, Hasel C, Adler G, Menke A (2006). Collagen type I induces disruption of E-cadherin-mediated cell-cell contacts and promotes proliferation of pancreatic carcinoma cells. Cancer Res.

[8] Hartel M, Di Mola FF, Gardini A, Zimmermann A, Di Sebastiano P, Guweidhi A, Innocenti P, Giese T, Giese N, Buchler MW, Friess H (2004). Desmoplastic reaction influences pancreatic cancer growth behavior. World J Surg.

[9] Cannon A, Thompson C, Hall BR, Jain M, Kumar S, Batra SK (2018). Desmoplasia in pancreatic ductal adenocarcinoma: insight into pathological function and therapeutic potential. Genes Cancer.

[10] Moffitt RA, Marayati R, Flate EL, Volmar KE, Loeza SG, Hoadley KA, Rashid NU, Williams LA, Eaton SC, Chung AH, Smyla JK, Anderson JM, Kim HJ (2015). Virtual microdissection identifies distinct tumor- and stroma-specific subtypes of pancreatic ductal adenocarcinoma. Nat Genet.

[11] Bachem MG, Schunemann M, Ramadani M, Siech M, Beger H, Buck A, Zhou S, Schmid-Kotsas A, Adler G (2005). Pancreatic carcinoma cells induce fibrosis by stimulating proliferation and matrix synthesis of stellate cells. Gastroenterology.

[12] Drifka CR, Eliceiri KW, Weber SM, Kao WJ (2013). A bioengineered heterotypic stroma-cancer microenvironment model to study pancreatic ductal adenocarcinoma. Lab Chip.

[13] Froeling FE, Mirza TA, Feakins RM, Seedhar A, Elia G, Hart IR, Kocher HM (2009). Organotypic culture model of pancreatic cancer demonstrates that stromal cells modulate E-cadherin, beta-catenin, and Ezrin expression in tumor cells. Am J Pathol.

[14] Ware MJ, Keshishian V, Law JJ, Ho JC, Favela CA, Rees P, Smith B, Mohammad S, Hwang RF, Rajapakshe K, Coarfa C, Huang S, Edwards DP (2016). Generation of an in vitro 3D PDAC stroma rich spheroid model. Biomaterials.

[15] Boj SF, Hwang CI, Baker LA, Chio II, Engle DD, Corbo V, Jager M, Ponz-Sarvise M, Tiriac H, Spector MS, Gracanin A, Oni T, Yu KH (2015). Organoid models of human and mouse ductal pancreatic cancer. Cell.

[16] Nicola NA, Babon JJ (2015). Leukemia inhibitory factor (LIF). Cytokine Growth Factor Rev.

[17] Albrengues J, Bertero T, Grasset E, Bonan S, Maiel M, Bourget I, Philippe C, Herraiz Serrano C, Benamar S, Croce O, Sanz-Moreno V, Meneguzzi G, Feral CC (2015). Epigenetic switch drives the conversion of fibroblasts into proinvasive cancer-associated fibroblasts. Nat Commun.

[18] Albrengues J, Bourget I, Pons C, Butet V, Hofman P, Tartare-Deckert S, Feral CC, Meneguzzi G, Gaggioli C (2014). LIF mediates proinvasive activation of stromal fibroblasts in cancer. Cell Rep.

[19] Bonan S, Albrengues J, Grasset E, Kuzet SE, Nottet N, Bourget I, Bertero T, Mari B, Meneguzzi G, Gaggioli C (2017). Membrane-bound ICAM-1 contributes to the onset of proinvasive tumor stroma by controlling acto-myosin contractility in carcinoma-associated fibroblasts. Oncotarget.

[20] Kamohara H, Ogawa M, Ishiko T, Sakamoto K, Baba H (2007). Leukemia inhibitory factor functions as a growth factor in pancreas carcinoma cells: Involvement of regulation of LIF and its receptor expression. Int J Oncol.

[21] Provenzano PP, Cuevas C, Chang AE, Goel VK, Von Hoff DD, Hingorani SR (2012). Enzymatic targeting of the stroma ablates physical barriers to treatment of pancreatic ductal adenocarcinoma. Cancer Cell.

[22] Barry-Hamilton V, Spangler R, Marshall D, McCauley S, Rodriguez HM, Oyasu M, Mikels A, Vaysberg M, Ghermazien H, Wai C, Garcia CA, Velayo AC, Jorgensen B (2010). Allosteric inhibition of lysyl oxidase-like-2 impedes the development of a pathologic microenvironment. Nat Med.

[23] Totaro A, Castellan M, Battilana G, Zanconato F, Azzolin L, Giulitti S, Cordenonsi M, Piccolo S (2017). YAP/TAZ link cell mechanics to Notch signalling to control epidermal stem cell fate. Nat Commun.

[24] Liu SC, Tsang NM, Chiang WC, Chang KP, Hsueh C, Liang Y, Juang JL, Chow KP, Chang YS (2013). Leukemia inhibitory factor promotes nasopharyngeal carcinoma progression and radioresistance. J Clin Invest.

[25] Bressy C, Lac S, Nigri J, Leca J, Roques J, Lavaut MN, Secq V, Guillaumond F, Bui TT, Pietrasz D, Granjeaud S, Bachet JB, Ouaissi M (2018). LIF Drives Neural Remodeling in Pancreatic Cancer and Offers a New Candidate Biomarker. Cancer Res.

[26] Biffi G, Oni TE, Spielman B, Hao Y, Elyada E, Park Y, Preall J, Tuveson DA (2018). IL-1-induced JAK/STAT signaling is antagonized by TGF-beta to shape CAF heterogeneity in pancreatic ductal adenocarcinoma. Cancer Discov.

[27] Conroy T, Desseigne F, Ychou M, Bouche O, Guimbaud R, Becouarn Y, Adenis A, Raoul JL, Gourgou-Bourgade S, de la Fouchardiere C, Bennouna J, Bachet JB, Khemissa-Akouz F (2011). FOLFIRINOX versus gemcitabine for metastatic pancreatic cancer. N Engl J Med.

[28] Hall BR, Cannon A, Atri P, Wichman CS, Smith LM, Kumar S, Batra SK, Wang H, Ganti AK, Sasson AR, Are C (2018). A Comparative Analysis of Survival and Funding Discrepancies in Cancers with High Mortality. Ann Surg.

[29] Neesse A, Michl P, Tuveson DA, Ellenrieder V (2014). nab-Paclitaxel: novel clinical and experimental evidence in pancreatic cancer. Z Gastroenterol.

[30] Liu WB, Wang XP, Wu K, Zhang RL (2005). Effects of angiotensin II receptor antagonist, Losartan on the apoptosis, proliferation and migration of the human pancreatic stellate cells. World J Gastroenterol.

[31] Hurwitz H, Van Cutsem E, Bendell J, Hidalgo M, Li CP, Salvo MG, Macarulla T, Sahai V, Sama A, Greeno E, Yu KH, Verslype C, Dawkins F (2018). Ruxolitinib + capecitabine in advanced/metastatic pancreatic cancer after disease progression/intolerance to first-line therapy: JANUS 1 and 2 randomized phase III studies. Invest New Drugs.

[32] Kumar S, Das S, Rachagani S, Kaur S, Joshi S, Johansson SL, Ponnusamy MP, Jain M, Batra SK (2015). NCOA3-mediated upregulation of mucin expression transcriptional and post-translational changes during the development of pancreatic cancer. Oncogene.

