# Supplementary material for: Utilizing cell line-derived organoids to evaluate the efficacy of a novel LIFR-inhibitor, EC359 in targeting pancreatic tumor stroma
Source: Genes Cancer. 2019 Feb;10(1-2):1–10. doi: 10.18632/genesandcancer.184 (PMC6420790; doi:10.18632/genesandcancer.184)
Supplement: Supplementary file 1 [file ganc-10-001-s001.pdf]

Utilizing cell line-derived organoids to evaluate the efficacy of a novel LIFR-inhibitor, EC359 in targeting pancreatic tumor stroma – Hall et al

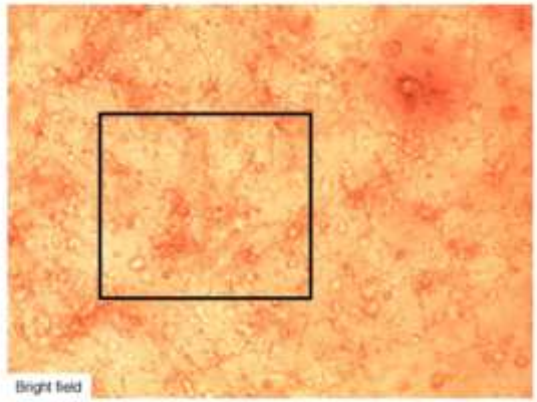

Supplementary Figure 1: Microscopic view of organoids after five days of 3D co-culture conditions demonstrating the formation of organized structures within the organoids. Magnification 200x

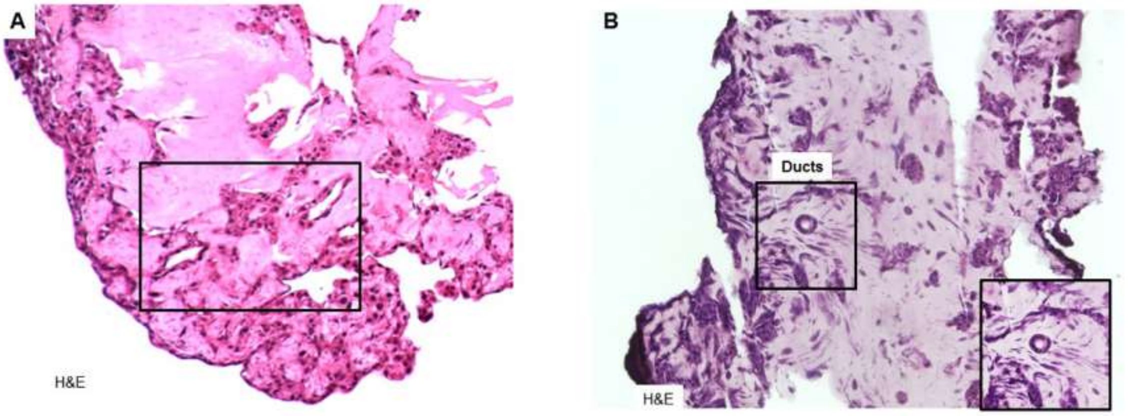

Supplementary Figure 2: A, B) Hematoxylin and Eosin tissue staining depicting the formation of pancreatic ducts surrounded by pancreatic stellate cells. Magnification 200x

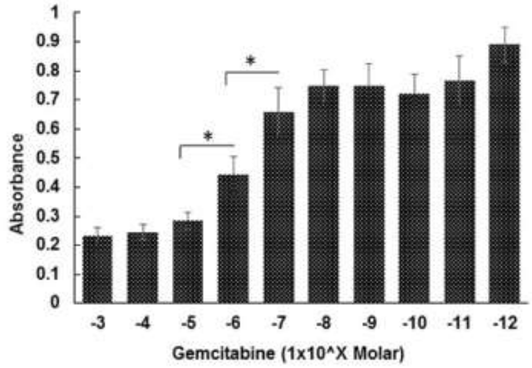

Supplementary Figure 3: The 3D *in vitro* MTT assay of organoids grown over the course of seven days and treated with varying concentration of gemcitabine on PIDs 4, 5, and 6. MTT assay performed on PID 7 demonstrated the IC50 of approximately 1uM for pancreatic cancer and stellate cells grown under 3D co-culture conditions. *p<0.05

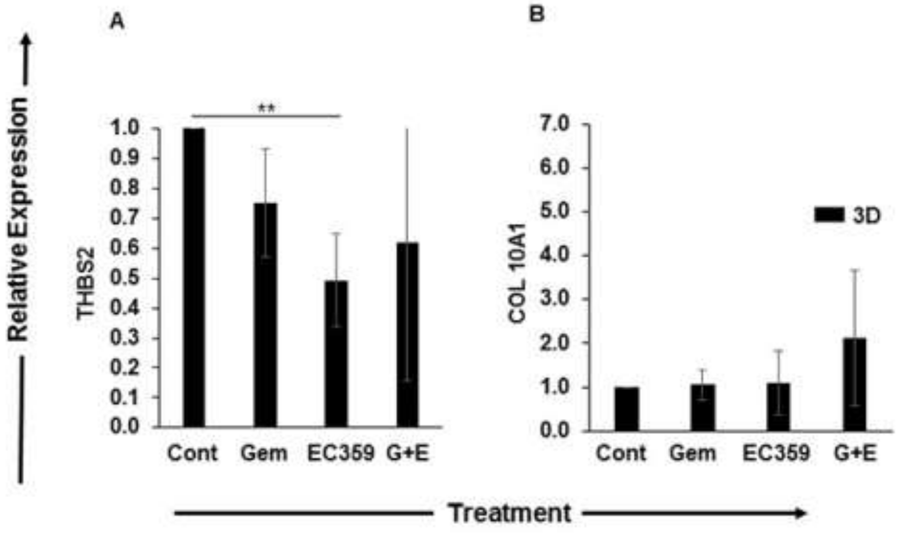

Supplementary Figure 4: The qRT-PCR analysis of markers of activated stroma, THBS2 and Collagen 10A1 (COL 10A1) after treatment of 3D organoids with gemcitabine (Gem), EC359, and combination of gemcitabine and EC359 (G+E) generated using pancreatic cancer and stellate cells. **p<0.001

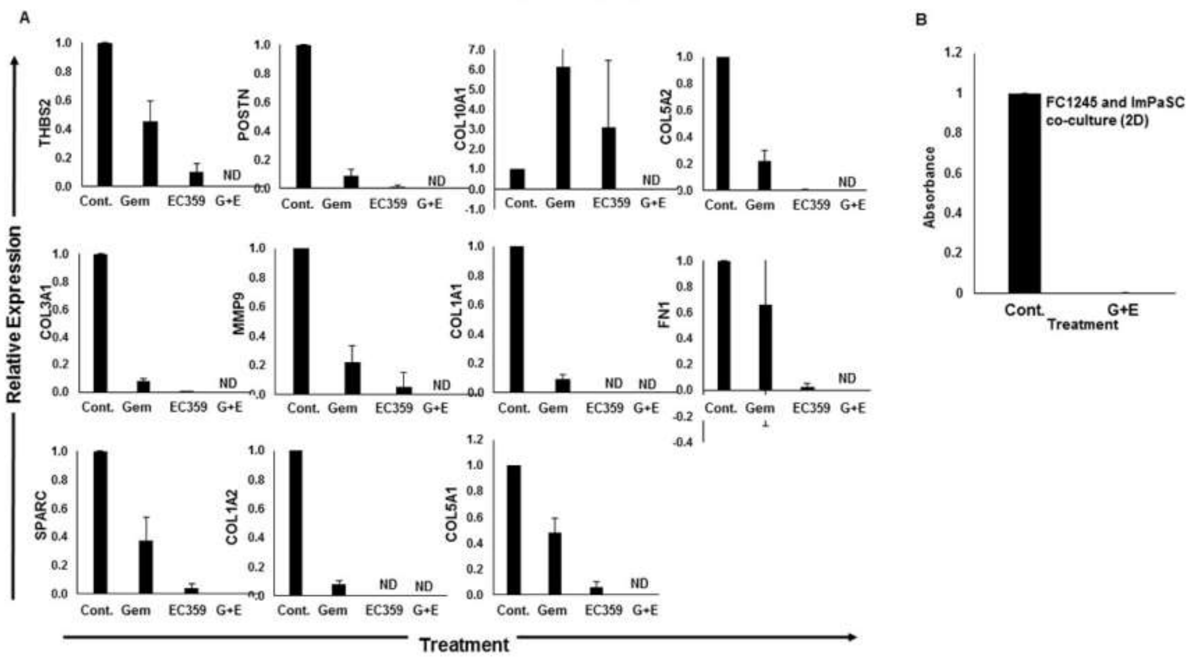

Supplementary Figure 5: A) The qRT-PCR analysis of markers of activated stroma (THBS2, POSTN, MMP9, SPARC, and FN1) and the collagen proteins after treatment with gemcitabine (Gem), EC359, and combination of gemcitabine and EC359 (G+E) under 2D co-culture conditions using pancreatic cancer (FC1245) and PSCs (ImPaSC) cells. **B)** MTT assay performed under 2D co-culture conditions using pancreatic cancer (FC1245) and PSCs (ImPaSC) cells demonstrated a significant toxicity by the combined treatment with EC359 and gemcitabine.
